# Novel drug research and therapeutic strategies targeting tumor metastasis and cancer stem cells

**DOI:** 10.3389/fphar.2025.1643183

**Published:** 2025-09-16

**Authors:** Sicong Xie, Zhiyi Zhou, Yu Zheng, Chenshuo Yu, Weihan Kong, Yushan Chen, Wenzhe Si, Fei Zhou, Zixuan Yang, Ruoxuan Ni, Cheng Chang, Yang Zhang

**Affiliations:** ^1^ Department of Rehabilitation Medicine, School of Acupuncture-Moxibustion and Tuina and School of Health Preservation and Rehabilitation, Nanjing University of Chinese Medicine, Nanjing, China; ^2^ Department of Cardiology, Kunshan Hospital of Traditional Chinese Medicine, Kunshan, China

**Keywords:** cancer metastasis, cancer stem cells, targeted therapy, nanoparticle delivery, drug resistance

## Abstract

Cancer metastasis and stem cells (CSCs) drive resistance and most cancer deaths. Novel agents like Thiolatia (PSMD14 inhibitor) suppress metastasis and enhance chemotherapy efficacy. Sulfarotene targets tumor-repopulating cells in liver cancer with low toxicity. PTC 209 utilizes the high affinity of modified hyaluronic acid nanoparticles for colorectal cancer to reverse CSC stemness in colorectal cancer. Platinum hybrids (HY1-Pt, Salvigenin-Pt) overcome resistance through dual mechanisms. Natural compound Cantharidin inhibits metastasis but requires toxicity optimization. These strategies emphasize specificity, nanodelivery, and combination therapies to reduce toxicity and resistance, highlighting precision oncology potential. Clinical validation remains critical for translation.

## 1 Introduction

Cancer is a heterogeneous disease composed of multiple cells characterized by abnormal cell growth and proliferation (Roy and Saikia, 2016). About 90% of cancer patients die from tumor metastasis (Torre et al., 2016; Wang et al., 2018), and therapeutic options to stop tumor metastasis include inhibiting neoangiogenesis, blocking epithelial mesenchymal transition and targeting metastasis suppressors (Gerstberger et al., 2023). Some of the drugs that have been applied in the clinic such as Denosumab (RANKL monoclonal antibody) was approved in 2018 for the prevention of multiple myeloma and bone metastases, which reduces bone metastasis by inhibiting osteoclast activity (Clézardin et al., 2021); bevacizumab (VEGF monoclonal antibody) is used in the treatment of colon cancer and lung cancer by inhibiting tumor angiogenesis (Garcia et al., 2020), etc., but the current drugs used in the treatment of cancer metastasis have problems such as high toxicity. However, the current drugs for cancer metastasis have problems such as high toxicity, easy drug resistance, etc. Therefore the development of new anti-tumor metastasis drugs is imminent.

Cancer stem cells (CSCs) are a key subpopulation in tumors with properties such as self-renewal, differentiation, invasion, and drug resistance that drive tumorigenesis, metastasis, and recurrence (Batlle and Clevers, 2017). CSCs have become one of the main causes of therapeutic failure by mediating chemotherapy resistance through mechanisms such as transport proteins and gene mutations (Nassar and Blanpain, 2016). Tumor metastasis and drug resistance are both dependent on CSCs, and their unlimited proliferative capacity and resistance to standard therapies are major challenges in current tumor therapy (Walcher et al., 2020). Therefore, targeted reversal of CSCs stemness may become an important research direction for the treatment of tumor metastasis.

Currently, advanced stage II cancers are usually only detected after multiple metastases have occurred. Challenges associated with detecting dormant cancer cells or small metastases further complicate cancer treatment. In addition, drugs targeting cancer metastasis typically exhibit high cytotoxicity, inconsistent patient outcomes, and lead to the development of drug resistance (Bagchi et al., 2021; Wang M. et al., 2021). Therefore, there is an urgent need to develop novel small molecules, biologic drugs, and combination therapies that target key processes in cancer metastasis (Cha et al., 2020; Walcher et al., 2020). For example, Nethi and Li et al. integrated epidermal growth factor receptor (EGFR) targeted antibodies into mesenchymal stem cells (MSCs) and used them in combination with paclitaxel nanoparticles for delivery. This significantly inhibited the growth of *in situ* A549 tumors and effectively improved overall survival rates (Nethi et al., 2023). This paper summarizes recent research advances in drugs, mechanisms and potential therapeutic targets against tumor metastasis and stemness. The main topics include: novel inhibitors such as Thiolatia (PSMD14 inhibitor), PTC 209 (BMI-1 inhibitor); new and improved specific compounds such as HY1-Pt, Salvigenin platinum (IV) complex; multi-targeting inhibitor CTD, and low-toxicity inhibitor WYC-209, as show in Table 1.

**TABLE 1:** Novel drug treatment pathways for tumor metastasis and cancer stem cells.

Names	Cancer types	Pathways	Structure Column
Thiolatia	Esophageal Squamous Cell Carcinoma	PSMD14/SNAIL	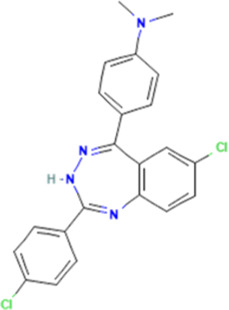
Terpenoid Cantharidin	Skin Cancer	PI3K/AKT/mTOR	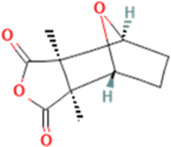
	Bladder Cancer	MAPK	
	Non-Small Cell Lung Cancer	Bcl2/Bax	
	Colorectal Cancer	JNK	
	Hepatocellular Carcinoma	NF-κB	
	Gastric Cancer	ERK	
	Cholangiocarcinoma	PKC	
	Breast Cancer	β-catenin	
	Pancreatic Cancer	Wnt/β-catenin	
	Oral Cancer	PI3/AKT	
Sulfarotene (WYC-209)	Hepatocellular Carcinoma	RARα-SOS2-RAS	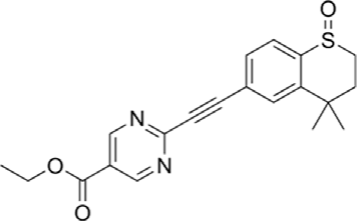
Sustained-release BMI-1 Inhibitor (PTC 209)	Colorectal Cancer	BMI-1	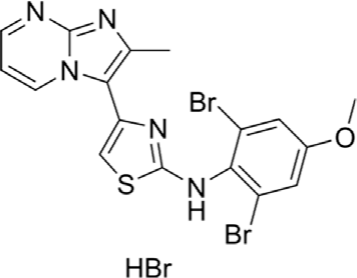
Novel CK2-Specific Pt (II) Compound: HY1-Pt	Non-Small Cell Lung Cancer	Wnt/β-catenin	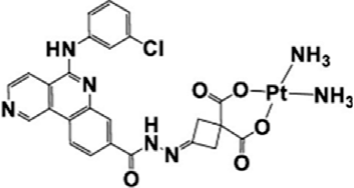
		Mitochondrial Apoptotic Pathway	
Salvigenin Ligand Platinum (IV) Complex	Esophageal Squamous Cell Carcinoma	Rap1b-mediated Signaling Pathway	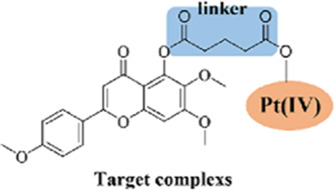
		Wnt/β-catenin Signaling Pathway	

## 2 Novel small molecules and drugs

### 2.1 Thiolatia: a PSMD14 inhibitor

Thiolatia (THL), a zinc chelator, is a disulfide-containing antibiotic and antiangiogenic compound. THL has been shown to inhibit adhesion to vitronectin and tumor-induced angiogenesis *in vivo* by decreasing paxillin in human umbilical vein endothelial cells (HUVECs) (Minamiguchi et al., 2001); and to block Hsp27 phosphorylation by inducing endothelial cell adhesion and wound/tumor-driven angiogenesis *in vitro* (Jia et al., 2010). Moreover, THL inhibits JAMM domain-containing proteases such as PSMD14 through catalytic Zn^2+^ ion complexes with the enzyme’s active center (Lauinger et al., 2017).

**FIGURE 1:**
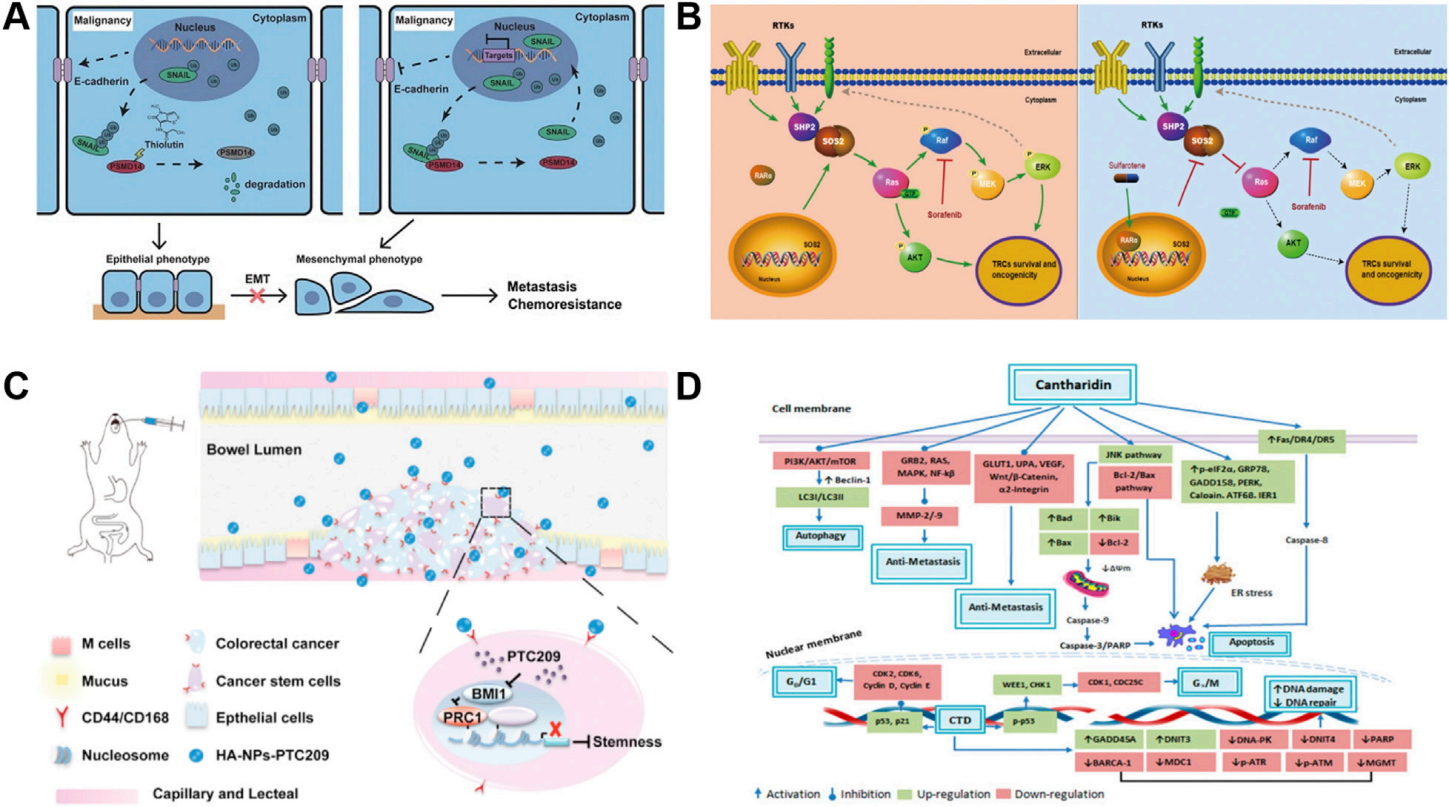
Mechanism of action of novel small molecules and drugs. **(A)** Schematic of the mechanism of THL for suppressing ESCC malignancy. Reproduced with permission from Jing et al. (2021). Image copyright belongs to Ivyspring International Publisher; no permission is required. **(B)** Schema depicting the mechanism by which sulfarotene targets the RAR SOS2-RAS signal axis to inhibit cancer cell growth and overcome drug resistance. Reproduced with permission from Qi et al. (2021), licensed under CC BY. **(C)** Proposed mechanism of HA-NPs-PTC209 action. After oral treatment, HA-NPs-PTC209 actively target CSCs in colorectal cancer (CRC) and are internalized. Consequently, the released PTC209 inhibits BMI-1 and downregulates the expression of stemness-re lated proteins to lower tumor stemness and recur rence. Reproduced with permission from Xu et al. (2019). Permission has been obtained from Elsevier. **(D)** Nticancer attributes of cantharidin and its molecular targets. Reproduced with permission from Naz et al. (2020), licensed under CC BY.

PSMD14 is highly expressed in a variety of cancers and acts as an oncogene to promote tumor development and progression (Zhu et al., 2018); it has been suggested that PSMD14 may be involved in esophageal squamous cell carcinoma (ESCC) tumorigenesis (Ma et al., 2016; Seo et al., 2019; Lv et al., 2020). As shown in Figure 1A that THL reverses the epidermal mesenchymal transition (EMT) process (Lamouille et al., 2014; Fischer et al., 2015; Zheng et al., 2015) (e.g., upregulation of E-Cadherin, inhibition of mesenchymal marker expression) by blocking PSMD14-mediated deubiquitination of SNAIL and decreasing the stability of SNAIL protein (Liu et al., 2017; Perez et al., 2017; Wu et al., 2017; Li et al., 2018). In addition, THL interferes with cytoskeletal reorganization and inhibits the formation of invagination cristae, which is essential for cell motility, weakening the motility and invasive ability of tumor cells. It also enhances the sensitivity of cancer cells to cisplatin, reducing side effects by reducing the dose of cisplatin, resulting in patient benefit (Jing et al., 2021).

Traditional proteasome inhibitors (e.g., bortezomib, carfilzomib) have more serious toxic side effects and limited efficacy in solid tumors compared with specific target inhibitors such as THL (Hideshima et al., 2005; Li J. et al., 2017; Song et al., 2017). In contrast, THL, as a PSMD14-specific inhibitor, is more selective and may be a safer anti-metastatic drug by precisely regulating the EMT and chemo-sensitivity-related pathways, combining both anti-metastatic and chemo-sensitizing effects (Deshaies, 2014; Jing et al., 2021).

### 2.2 Sulfarotene (WYC-209)

Sulfarotene (WYC-209) is advantageous in that it possesses negligible toxicity and highly selective inhibition of the growth and tumor-inducing ability of tumor reconstructive cells (TRCs) (Jiang et al., 2020) in various types of cancers. It is believed that sulfarotene blocks the activation of pro-tumorigenic signals downstream of RAS by upregulating retinoic acid receptor α (RARα) in hepatocellular carcinoma (HCC) TRCs and inhibiting the expression of SOS2 (Liceras-Boillos et al., 2018), a key mediator of the RAS signaling pathway (Sheffels et al., 2018; Schwartz et al., 2019; Wołoszynowska-Fraser et al., 2020) (Figure 1B). This pathway not only drives the self-renewal and tumorigenicity of TRCs (Li et al., 2015), but is also closely associated with resistance to drugs such as sorafenib. In multiple preclinical models, sulfarotene demonstrated efficient and selective inhibition of HCC TRCs (Qi et al., 2021).

Traditional targeted drugs (e.g., sorafenib) are limited in their clinical application due to tumor cell stemness-induced resistance and their inherent toxicity (Wei et al., 2019; Wang et al., 2020; Xia et al., 2020). By precisely targeting the RARα-SOS2-RAS axis and directly interfering with the stemness maintenance and resistance mechanisms of TRCs, sulfarotene not only overcomes the limitations of existing drugs, but also demonstrates a potent inhibitory effect on metastatic foci. It provides a highly promising therapeutic strategy to improve the prognosis of HCC patients (Qi et al., 2021).

### 2.3 Sustained release BMI 1 inhibitor (PTC 209)

Many anticancer drugs belong to the class IV of the Biopharmaceutical Classification System (BCS), which comprises substances with both low solubility in aqueous fluids and low apparent permeability. The high recurrence and metastasis of colorectal cancer (CRC) (Todaro et al., 2010) are often attributed to the maintenance of stemness in cancer stem cells (CSCs) (Batlle and Clevers, 2017), whose self-renewal, drug-resistant, and invasive properties lead to therapeutic failures (Zhang Z. et al., 2016). BMI-1 (B-cell-specific Moloney Murine Leukemia Virus Integration Site 1), as a key regulator of the stemness of CSCs (Kreso et al., 2014), is overexpressed in CRC and correlates with tumor progression and poor prognosis (Siddique and Saleem, 2012). Inhibition of BMI-1 can reverse the stemness of CSCs and has been applied to the treatment of colon cancer, myeloma and acute myeloid leukemia (Mourgues et al., 2015). Based on this, the investigators developed PTC209, a specific inhibitor against BMI-1 (Bolomsky et al., 2016).

To solve the problems of poor solubility, complex gastrointestinal environment and non-specific distribution faced by oral drug delivery (Mitragotri et al., 2014), the researchers developed a targeted delivery system based on poly (ethylene glycol)-poly (hydroxyglycolic acid) lactate (PEG-PLGA) nanoparticles (Wang et al., 2011; Mazzaferro et al., 2013; Lin et al., 2018). The nanoparticles were synthesized by a double emulsion method and modified with hyaluronic acid (HA) as a CD44/CD168-targeting ligand (HA-NPs-PTC209) (Huang et al., 2014; Choi et al., 2019; Leng et al., 2019), which significantly enhanced the targeting ability of the BMI-1 inhibitor (Zhang M. et al., 2016). The results of the *in vivo* antitumor experiments showed that HA-NPs-PTC209 significantly inhibited the growth and metastasis of CT26 orthogonal xenografts, which led to the *in situ* colon tumor accumulation in in situ colon tumors, thus reversing CSC stemness. The high *in vitro* stability of this targeted nano-example and the high permeability of the drug through the intestinal barrier offer the possibility of mitigating systemic adverse effects and improving therapeutic efficiency (Xu et al., 2019) (Figure 1C).

### 2.4 Novel CK2-specific platinum (II) compounds: HY1-Pt

The high mortality rate of non-small cell lung cancer (NSCLC) is closely related to chemoresistance and metastasis mediated by cancer stem cells (CSCs) (Martins-Neves et al., 2018). Conventional platinum-based drugs (e.g., cisplatin) have limited efficacy due to the DNA damage repair ability and drug resistance of CSCs (Nagasaka and Gadgeel, 2018; Wang et al., 2019). The CK2 inhibitor HY1 was found to have a strong inhibitory effect on CSCs in A549 cells (Schwind et al., 2017). Taking advantage of the inherent CK2 specificity and CSC inhibition of HY1, by conjugating HY1 with an active platinum (II) unit (Czarnomysy et al., 2018), the researchers developed a novel CK2-specific platinum (II) compound, HY1-Pt, which achieves the reversal of drug resistance and the inhibition of CSCs through the synergistic effect of targeting protein kinase CK2 with platinum-based drugs (Purwin et al., 2016). It was demonstrated that HY1-Pt specifically inhibited CK2 activity, blocked its mediated stemness-promoting signaling pathways such as Hedgehog/Gli1 and Wnt/β-catenin, and downregulated the expression of CSCs markers (e.g., Nanog, Oct-4) (Lu et al., 2013; Wang et al., 2017). Furthermore, HY1-Pt could enhance platinum drugs-induced DNA damage by interfering with the phosphorylation of DNA repair proteins by CK2 to inhibit the tumor cell repair ability. *In vitro* experiments demonstrated that HY1-Pt showed potent cytotoxicity (IC_50_ significantly lower than cisplatin) and selectively inhibited the formation of CSCs spheroids in A549/cDDP cells and in the A549/cDDP xenograft model, HY1-Pt significantly inhibited the tumor growth without triggering significant toxicity reactions.

Existing CK2 inhibitors (e.g., CX-4945) have limited clinical efficacy, while conventional platinum drugs are limited by drug resistance and toxicity. HY1-Pt’s synergistic effect through a “dual-targeting” strategy (CK2 inhibition + platinum DNA damage) is expected to improve the prospects for platinum-based therapies and to reverse the resistance to cisplatin (Wang et al., 2021b).

### 2.5 Salvigenin ligand platinum (IV) complexes

The high invasiveness and cisplatin resistance of esophageal squamous cell carcinoma (ESCC) are closely related to the maintenance of stemness in cancer stem cells (CSCs) (Najafi et al., 2019; Mao et al., 2021). The expression level of RAS-associated protein 1b (Rap1b), as a member of the RAS superfamily, has been confirmed to correlate positively with stemness of CSCs, and overexpression of Rap1b in ESCC positively regulates CSC proliferation, invasion, and stemness, making it a novel target for reversing ESCC drug resistance (Noguchi et al., 2015; Jia et al., 2017; Zhang et al., 2019). Guo et al. found that Rap1b was overexpressed in glioma stem cells (GSCs), and silencing Rap1b could effectively inhibit the growth and invasion of glioma cells (Guo et al., 2023). In addition, Rap1b has been shown to promote hematopoietic stem cell development by enhancing integrin-mediated cell adhesion (Rho et al., 2019).

Based on this, researchers have developed novel platinum (IV) complexes (e.g., complex-1) (Zhang et al., 2017) with the natural polyphenolic compound Salvigenin as a ligand to inhibit Rap1b and overcome cisplatin resistance through a dual mechanism. Complex-1, which consists of a platinum (IV) core conjugated with Salvigenin ligand, significantly downregulates cancer cell stemness by inhibiting Rap1b expression and blocking its mediated integrin signaling and Wnt/β-catenin/TCF pathway; and enhances the platinum accumulation in cisplatin-resistant cells (TE6/cDDP) and inhibits the DNA damage repair ability (Fang et al., 2019).

As the first platinum (IV) complex that potently inhibits Rap1b and effectively reverses cisplatin-induced drug resistance, complex-1 fills the gap that there is no effective solid molecule inhibitor for Rap1b, and provides a new way of thinking for the development of Rap1b inhibitors and overcoming cisplatin-induced drug resistance in cancer cells (Zhao et al., 2024).

### 2.6 Terpenoid cantharidin (CTD)

Cantharidin (CTD), a terpenoid isolated from blister beetles and used in traditional Chinese medicine for the treatment of a variety of diseases and cancers (Deng et al., 2013), has been shown to be an inhibitor of protein phosphatase 2A (PP2A) and heat shock transcription factor 1 (HSF-1), both of which are potential anticancer targets (Kim et al., 2013; Li W. et al., 2017). As shown in Figure 1D, CTD significantly inhibits the proliferation of a variety of solid tumors and leukemia cells, including liver cancer, pancreatic cancer, and colon cancer, by inhibiting PP1/PP2A activity, inducing apoptosis, interfering with cell cycle arrest and autophagy (Wu et al., 2014; Shen et al., 2015). By inhibiting PP1/PP2A activity, inducing apoptosis, interfering with protein synthesis, and triggering cell cycle arrest and autophagy, CTD significantly inhibits the proliferation of hepatocellular carcinoma, pancreatic carcinoma, colon carcinoma, and other solid tumors as well as leukemia cells (Huang et al., 2011; Hsia et al., 2015b; Ji et al., 2015; Su et al., 2015; Su et al., 2016; Liu et al., 2018). In addition, CTD can reduce the expression of DNA damage repair-related proteins, enhance the sensitivity of cancer cells to radiotherapy, and alleviate the sequelae of chemotherapy in combination therapy (Kuo et al., 2015; Xu et al., 2018).

Studies have demonstrated that CTD inhibits cancer cell invasion and migration by targeting and regulating metastasis-related signaling pathways in a variety of cancer cells. For example, in gastric cancer, CTD inhibited migration by down-regulating the CCAT1-mediated PI3K/AKT pathway (Song et al., 2020); in bladder cancer cells, it blocked cell adhesion and invasion by inhibiting the p38/JNK1/2 MAPK pathway and decreasing the enzyme activity and expression of MMP-2/9. In addition, CTD inhibited metastatic potential by inhibiting the PI3K/AKT/mTOR and NF-κB pathways and reducing UPA protein and matrix metalloproteinase activities in lung cancer models (Hsia et al., 2016).

CTD can effectively inhibit metastasis in different kinds of cancer cells; and the action of CTD involves key pathways such as MAPK, Bcl2/Bax, Wnt/β-catenin, ERK, etc., which inhibit tumor growth and metastasis through cross-regulation (Wang et al., 2015; Gu et al., 2017; Chun et al., 2018). Among them, PI3K/AKT/mTOR and MAPK pathways have been widely proved to be the core targets of CTD against tumor metastasis, and CTD, as a multi-targeted anticancer agent, has demonstrated its unique advantages in inhibiting tumor growth, metastasis, and synergistic radiotherapy. However, the toxicity of CTD itself remains to be solved (Hsia et al., 2015a; Naz et al., 2020).

## 3 Summary

Cancer metastasis and stemness maintenance of cancer stem cells (CSCs) are central causes of tumor treatment failure. About 90% of cancer patients die from metastasis, and the self-renewal, drug-resistant and invasive properties of CSCs drive tumor recurrence and spread (Torre et al., 2016; Wang et al., 2018). In recent years, significant progress has been made in the study of novel drugs and targets against CSCs and key pathways of metastasis, providing a new direction to break through the therapeutic bottleneck. We analyze and summarize the emergence of novel drugs and targets with potential translational ability against tumor metastasis and CSCs. Specific inhibitors serve as one of the hotspots for novel drug development by virtue of their low toxicity and high therapeutic efficiency.

New inhibitors such as Thiolatia (PSMD14 inhibitor) selectively inhibit the PSMD14 gene, which is highly expressed in many cancers, and its inhibition of key oncogenes ensures its efficacy in tumor metastasis; secondly, by virtue of its high specificity, it avoids the toxicity and side-effects of the traditional proteasome inhibitors; and the combination of the drug and cisplatin sensitization effect also gives it a wider scope of application. The combined sensitizing effect of the drug and cisplatin also gives it a wider scope of application (Deshaies, 2014; Jing et al., 2021). Sulfarotene, a sulfonamide drug, also has high selectivity for the target, inhibits RAS signaling pathway with high selectivity, blocks downstream oncogenic signals, and intervenes in the dry maintenance of TRCs; its low toxicity is also better than that of traditional drugs, and it is a drug with great therapeutic potential for cancer patients (Qi et al., 2021). The BMI 1 inhibitor (PTC 209) solves the problem of non-specific distribution of colorectal cancer drugs by using nanoparticles for targeted delivery, which enhances the permeability of the drug to the intestinal barrier. PTC 209 maintains the effect of the inhibitor while allowing the drug to accumulate in the *in situ* colon tumors, which effectively avoids the systemic adverse reactions of the patients and improves the therapeutic efficiency (Xu et al., 2019).

Platinum drugs such as cisplatin treat cancer by interfering with DNA and hindering the cell cycle. However, their non-specific therapeutic characteristics are characterized by problems such as drug resistance and toxic side effects (Nagasaka and Gadgeel, 2018; Wang et al., 2019). Current research has changed the therapeutic limitations of traditional platinum drugs by changing the chemical structure of platinum drugs and adding new ligands to form complexes. For example, HY1-Pt is a CK2 inhibitor HY1 conjugated to an active platinum (II) unit, which achieves resistance reversal and CSCs inhibition through the synergistic effect of targeting protein kinase CK2 and platinum drugs. This breaks through the limited clinical efficacy of existing CK2 inhibitors and significantly improves the resistance and toxicity of platinum drugs (Wang et al., 2021b). Some researchers have also used Salvigenin, a natural polyphenolic compound, as a ligand for novel platinum (IV) complexes, to potently inhibit Rap1b and effectively reverse cisplatin-induced drug resistance, filling the gap of no effective solid molecular inhibitor for Rap1b (Zhao et al., 2024). It provides a new idea to overcome cisplatin-induced drug resistance in cancer cells. The in-depth exploration of traditional drugs also continues, such as the terpenoid Cantharidin (CTD), a multi-targeted anticancer agent derived from traditional Chinese medicine, which plays an important role in inhibiting tumor proliferation and metastasis, enhancing sensitivity and mitigating side effects in combination with radiotherapy by inhibiting the activity of PP1/PP2A and modulating the pathways of PI3K/AKT/mTOR and MAPK. It plays an important role in inhibiting tumor proliferation and metastasis, enhancing sensitivity and alleviating side effects in combined radiotherapy (Hsia et al., 2015a; Naz et al., 2020). The toxicity problem may be solved in the future by nano-targeted delivery and structural modification optimization to enhance clinical safety. The above drugs have shown good therapeutic potential for tumor stemness in cell and animal experiments, and some of them have good performance in combining with sensitized classical anticancer drugs. Currently, some preclinical and clinical trials have demonstrated the efficacy of these drugs (Table 2). However, the potential toxicity, drug resistance, and clinical translation issues of these drugs still need to be solved.

**TABLE 2:** Preclinical or clinical data on novel drugs.

Names	Cancer types	Preclinical/clinical Data	Ref
Thiolutin	Esophageal squamous cell carcinoma	THL injection around the tumor inhibited tumor growth in ESCC xenografts in mice	Jing et al. (2021)
	Breast cancer	In endocrine resistant models, thiolutin could de-stabilize the resistant form of ERα (Y537S) and restore tamoxifen sensitivity	Yang et al. (2024)
Terpenoid Cantharidin	Breast cancer	Breast cancer patients undergoing postoperative chemotherapy who received sodium cantharidin injections had lower rates of leukopenia and gastrointestinal reactions than the control group	Wang et al. (2014)
	Prostate cancer	Cantharidin treatment can inhibit tumor cell proliferation and induce autophagy. Combination therapy with cantharidin and TRAIL may be a successful strategy for treating TRAIL-resistant prostate cancer	Nazim et al. (2020)
Sulfarotene (WYC-209)	Melanoma	WYC-209 eliminated 87.5% of melanoma tumor-repopulating cells (TRCs) in lung metastases in immunocompetent wild-type C57BL/6 mice at a dose of 0.22 mg/kg, without exhibiting significant toxicity	Chen et al. (2018)
	Liver cancer	WYC-209 effectively inhibited CSC resistance and terminated tumor growth and lung metastasis in mice without significant side effects	Qi et al. (2021)
Sustained-release BMI-1 Inhibitor (PTC 209)	Multiple myeloma	PTC-209 demonstrates potent anti-multiple myeloma activity by targeting core survival genes in multiple myeloma (such as MYC and MCL-1), inhibiting angiogenesis and osteoclast formation	Bolomsky et al. (2016)
	Primary liver cancer	Primary liver cancer mice showed significant improvement in liver function after treatment with PTC-209. This was achieved by inhibiting tumor proliferation and the expression of liver cancer CSCs *in vivo*	Li et al. (2023)
	Colorectal cancer	PTC 209 effectively inhibited tumor growth and reduced stem cell marker expression in mice with colon cancer. It significantly prevented metastasis to the gastrointestinal system and did not exhibit acute side effects	Xu et al. (2019)
Novel CK2-Specific Pt (II) Compound: HY1-Pt	Non-small Cell Lung Cancer	HY 1-Pt demonstrated effective *in vivo* antitumor activity in A549 and A549/cDDP mouse xenograft models, overcoming cisplatin resistance and exhibiting low toxicity	Wang et al. (2021b)
	Ovarian cancer	Intravenous administration of HY1-Pt effectively inhibits tumor growth in preclinical mouse models of A2780 and A2780/CDDP cells. Furthermore, high doses of HY1-Pt do not affect mouse body weight	Wang et al. (2023)
Salvigenin Ligand Platinum (IV) Complex	Esophageal squamous cell carcinoma	In the TE 6/cDDD transplant mouse model, Complex-1 showed strong ability to reverse cisplatin-induced cancer cell resistance and inhibit tumor growth, with a tumor growth inhibition rate of 73.3% at 13 mg/kg, and no significant systemic toxicity	Zhao et al. (2024)
	Gastric cancer	*In vivo* studies demonstrated that the enhanced accumulation of complex 14 contributed to tumor inhibition of 75.6% in SGC-7901/CDDP xenografts, which was much higher than cisplatin (25.9%) and oxaliplatin (43%)	Cao et al. (2021)

Currently, novel anticancer drugs targeting tumor metastasis and stem cells are characterized by high selectivity, optimized target delivery system, overcoming drug resistance and combination therapy, which provide diversified strategies to improve cancer prognosis. Further clinical validation and mechanism analysis will promote the arrival of the era of precision therapy.
